# Periphyton closes the nitrogen budget gap in rice paddies

**DOI:** 10.1093/nsr/nwag016

**Published:** 2026-01-13

**Authors:** Pengfei Sun, Yonghong Wu, Yin Chen, Jan Dolfing, Bruce E Rittmann, Kees Jan van Groenigen

**Affiliations:** State Key Laboratory of Soil and Sustainable Agriculture, Institute of Soil Science, Chinese Academy of Sciences, Nanjing 211135, China; College of Nanjing, University of Chinese Academy of Sciences, Nanjing 211135, China; School of Life Sciences, University of Warwick, Coventry CV4 7AL, UK; State Key Laboratory of Soil and Sustainable Agriculture, Institute of Soil Science, Chinese Academy of Sciences, Nanjing 211135, China; College of Nanjing, University of Chinese Academy of Sciences, Nanjing 211135, China; School of Life Sciences, University of Warwick, Coventry CV4 7AL, UK; School of Biosciences, The University of Birmingham, Birmingham B15 2TT, UK; Faculty of Energy and Environment, Northumbria University, Newcastle upon Tyne NE1 8QH, UK; Biodesign Swette Center for Environmental Biotechnology, Arizona State University, Tempe, AZ 85287, USA; Department of Geography, Faculty of Environment, Science and Economy, University of Exeter, Exeter EX4 4QJ, UK

**Keywords:** periphyton, nitrogen mass balance, paddy fields, nitrogen sequestration, precision nitrogen management

## Abstract

Persistent 4%–22% gaps in nitrogen (N) mass balances have hindered sustainable N management in paddy agriculture. Periphyton are known N sinks, yet their role in paddies remains unclear. We used ^15^N tracing in 840 paddies across China to quantify periphyton-associated N pools and their fate. Periphyton captured 6%–24% (mean: 12%) of the applied N fertilizer (i.e. ∼0.8 Tg N yr^−1^ nationwide), effectively accounting for the missing N in previous budgets. Most of the sequestered N was stored as bioavailable ammonium. Partitioning analysis revealed that periphyton-mediated N was subsequently released into residual soil N (512–640 kt), denitrification (56–128 kt) and ammonia volatilization (64–232 kt). Thus, periphyton act as transient N reservoirs, immobilizing N fertilizer early in the growing season and gradually releasing it through biomass decay. This overlooked pathway closes a critical gap in agroecosystem N cycling and supports more precise N management in rice systems.

## Teaser

Periphyton, microbial aggregates at the soilwater interface, temporarily sequesters fertilizer nitrogen in rice paddies. This periphyton-mediated nitrogen cycling helps explain the long-standing `missing nitrogen' in fertilizer budgets and opens new opportunities to improve nitrogen-use efficiency.

## INTRODUCTION

Rice is a staple food for more than half the world’s population [1]. Ensuring sustainable rice production is vital for stabilizing the global food supply, especially during times of crisis [2,3]. Nitrogen (N) presents a critical paradox in rice agriculture: although it is indispensable for achieving high yields [4], it is the leading contributor to agricultural pollution when mismanaged [5,6]. In China, the application of synthetic N fertilizer has increased by 170% since the 1960s [7,8]. This continued reliance on high external N inputs with persistently low N-use efficiency has led to a loss of ∼47% of applied N, resulting in severe environmental consequences [9–11]. Addressing the dual challenge of enhancing rice productivity and safeguarding ecological integrity requires precise N management grounded in a mechanistic understanding of N cycling within agroecosystems [12,13].

Systematic N-fertilizer budgeting, by resolving all N-flux pathways, is essential for optimizing nutrient-use efficiency and guiding policy frameworks for sustainable intensification [14]. However, substantial uncertainty remains surrounding N-transformation pathways in flood rice systems [15,16]. This uncertainty is reflected in global syntheses that consistently show that a significant portion (4%–22%) of applied N is not quantitatively accounted for [17–31]. This presents a key limitation in current N-cycling models and budgeting approaches [32]. Understanding the biogeochemical fate of this missing N is a scientific priority because resolving it is essential for developing effective mitigation strategies that align food-security goals with environmental sustainability [4,33,34].

As key players in biogeochemical cycles, microbial aggregates function as essential biogeochemical engines in natural ecosystems [35,36]. One type of microbial aggregate at the soil–floodwater interface (not within the soil), periphyton, has been largely overlooked in N-cycle research. Periphyton are millimeter-thick layers consisting of prokaryotes, eukaryotes and extracellular polymeric substances [37–40]. Building on prior evidence that periphyton can sequester N [41], we hypothesized that periphyton acts as a critical, previously unrecognized contributor to missing N fluxes in paddy fields.

To test this hypothesis, we conducted a large-scale investigation of 840 paddy fields from 2016 to 2019. Together, these sites cover >93% of China’s rice-cultivation area (see ‘Methods’). This was complemented by three on-farm ^15^N-isotope-tracing experiments across temperate, subtropical and tropical agroecological zones to enable the real-time tracking of N fluxes. Our findings revealed that periphyton mediates 6%–24% of the applied N fertilizer, directly accounting for the missing fraction in paddy-field N budgets. This microbial reservoir is rich in bioavailable N and is subsequently partitioned into four primary sinks: soil residual N (23%–37%), denitrification (2%–3%), ammonia volatilization (2%–26%) and temporary storage in periphyton biomass (16%–81%). This discovery marks a paradigm shift, positioning periphyton as a key N regulator that enhances N retention, while offering a novel leverage point for sustainable rice production.

## RESULTS

### National-scale quantification of periphyton N sequestration

**Figure 1.**
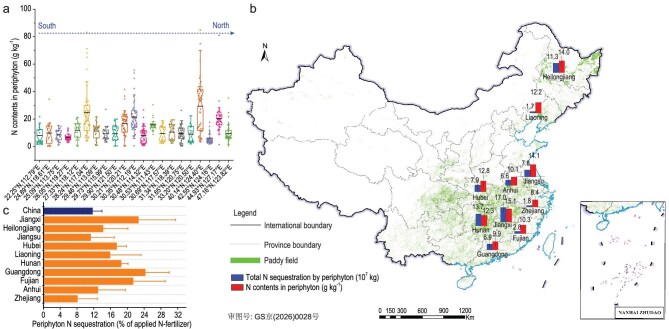
Nitrogen sequestration in periphyton across Chinese paddy fields. (a) N contents in periphyton collected from 840 sites, distributed across 21 sampling regions. The data points for each region represent the N contents of periphyton collected from different sampling sites within that region from 2016 to 2019. Black lines represent average values for each sampling region. (b) Average N contents and gross N loadings in periphyton in paddy fields at the provincial scale. (c) Proportion of N in periphyton to the average N-fertilizer input for rice production at provincial and national scales.

Periphyton showed substantial N-sequestration capacity, with intracellular N contents ranging from 0.38 to 34.49 g kg^−1^ across individual samples (Fig. 1a), indicating significant variation across sites. When aggregated at the provincial scale, these concentrations converged to a narrower range of 6.9 ± 0.9 to 15.1 ± 1.6 g kg^−1^, reflecting regional consistency in N-retention potential under shared climatic and agronomic conditions (Fig. 1b).

Quantification of periphyton-mediated N flux (Equations S1–S3) revealed province gross annual loads ranging from (1.7 ± 0.8) × 10^7^ kg in temperate Liaoning to (17.0 ± 6.6) × 10^7^ kg in subtropical Jiangxi (Fig. 1b). When expressed as percentages of province-specific N-fertilizer inputs (Table S1), these loads accounted for 6%–24% of the inputs (Fig. 1c). National-scale extrapolations using weighted averaging (Equations S4–S6) yielded an estimated average N-retention capacity of 25.9 kg N ha^−1^, equivalent to ∼0.8 teragrams of N sequestered annually, which is ∼12% of the total N fertilizer applied in China’s total rice production (Fig. 1c and Equation S7).

### Cross-climatic on-farm experimental validation

**Figure 2.**
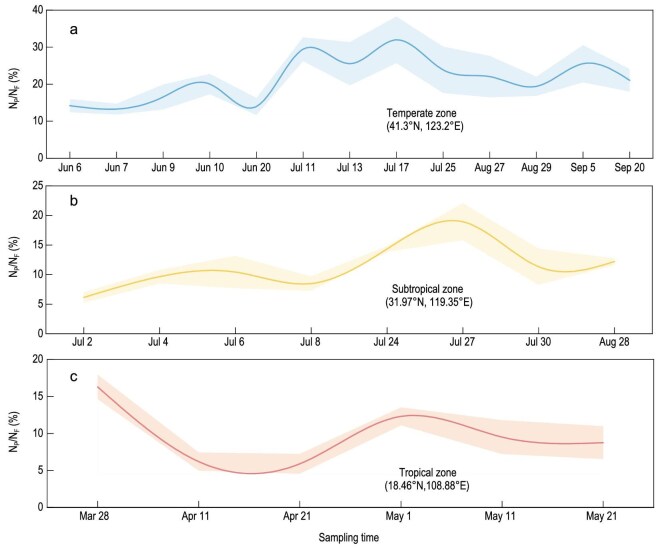
Temporal variation in the percentage of fertilizer N sequestered by periphyton (N_P_/N_F_ (%)) over the entire biofilm life cycle, from colonization (5 days post basal fertilizer application) and formation to apoptosis, at three on-farm experimental sites representing distinct climate zones. (a) Temperate zone (Shenyang) with a mean of 21.3% ± 3.8%. (b) Subtropical zone (Jurong), with a mean of 11.4% ± 1.6%. (c) Tropical zone (Ledong), with a mean of 9.3% ± 1.6%.

The N-sequestration capacity of periphyton was experimentally validated by using three ^15^N-isotope-tracing campaigns conducted across China’s rice-growing climate zones (temperate, subtropical and tropical). In temperate paddies (Shenyang, Liaoning), periphyton accounted for 21.3% ± 3.8% of the applied N (range: 14.2% ± 2.3% to 32.0% ± 6.3%; Fig. 2a). Subtropical systems (Jurong, Jiangsu) exhibited moderate control (11.4% ± 1.6%; range: 6.2% ± 0.8% to 18.9% ± 3.1%; Fig. 2b), consistently with the hydrological variability associated with monsoonal patterns that limit biofilm development. In tropical paddies (Ledong, Hainan), periphyton exerted a reduced yet stable influence (9.3% ± 1.6%; range: 5.9% ± 1.3% to 16.3% ± 1.7%; Fig. 2c). Averaged across the growing season and across sites, 14% of the applied N fertilizer was incorporated into the periphyton biomass (Equation S8)—a value closely aligned with those of national-scale survey estimates (12%, Fig. 1c).

**Figure 3.**
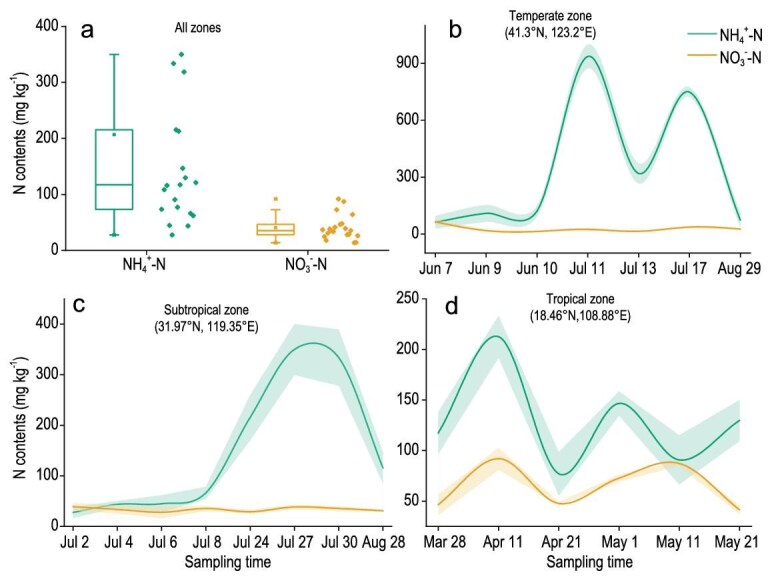
Nitrogen forms in periphyton from rice paddies across three climatic zones. (a) Comparison of NH_4_^+^–N and NO_3_^−^–N contents in periphyton from all zones combined. (b–d) Temporal variation across the entire periphyton life cycle in NH₄⁺–N and NO_3_⁻–N contents in periphyton from the (b) temperate, (c) subtropical and (d) tropical zones, respectively.

Our field experiments further demonstrated a clear dominance of bioavailable ammonium (NH_4_⁺–N) as the primary N form stored in periphyton. At all three sites, the NH_4_⁺–N contents consistently exceeded those of nitrate (NO_3_⁻–N) by at least an order of magnitude (Fig. 3a). Ammonium accumulation followed a distinct climate-driven unimodal pattern, peaking in the temperate zone (935.7 ± 63.1 mg kg^−1^; Fig. 3b), with progressively lower levels in the subtropical (349.8 ± 50.3 mg kg^−1^; Fig. 3c) and tropical (212.6 ± 20.7 mg kg^−1^; Fig. 3d) zones.

**Figure 4.**
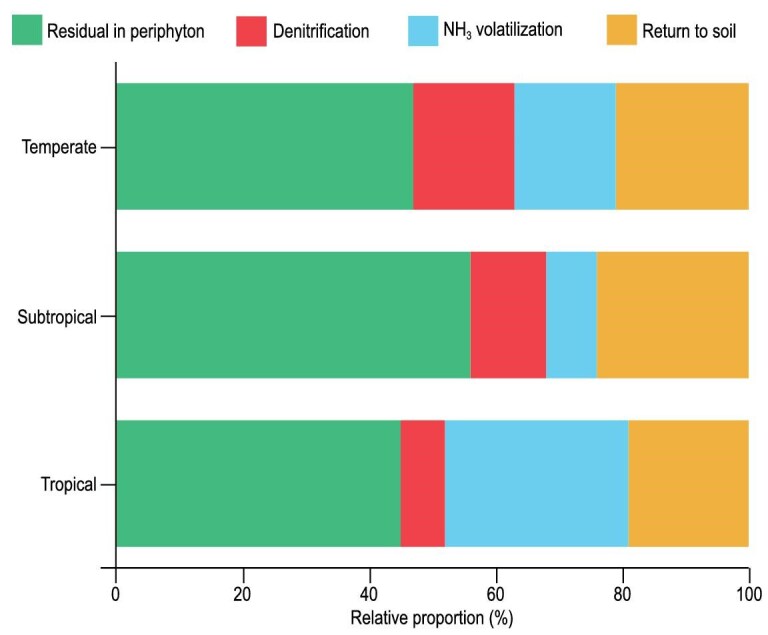
Fates of intercellular N in periphyton obtained from three ^15^N-tracing experiments across three rice-planting climate zones.

We used ^15^N partitioning to quantify the four primary fates of periphyton-sequestered N across the rice growth period (Fig. 4): return to soil (range: 19%–24%; mean: 21%), NH_3_ volatilization (8%–29%; mean: 18%), denitrification-derived N_2_O gaseous loss (7%–16%; mean: 12%) and transient retention in periphyton biomass (mean: 45%–56%; 49%), with the latter ultimately contributing to the soil N pool.

We estimated the proportional distribution of N sequestered by periphyton in China among distinct biogeochemical pathways by multiplying the total sequestered N pool (0.8 Tg) by the empirically derived fractional allocation coefficients for each fate. Using this approach, we estimated that 152–192 kilotonnes (kt; mean: 171 kt) of N are released into paddy soils during periphyton growth, while 360–448 kt (mean: 395 kt) are returned to the system post-harvest through tillage, resulting in a total of 512–640 kt (mean: 565 kt) of N that are finally returned to the soil. N loss occurs via ammonia volatilization (64–232 kt; mean: 141 kt) and denitrification (56–128 kt; mean: 93 kt). These results define periphyton as a significant N sink in China, thereby setting the stage for examining its broad biogeochemical and management implications.

## DISCUSSION

The transformation of agricultural N management from pollutant-emitting linear flows to circular low-loss systems represents a cornerstone of planetary sustainability [9–11]. Our results showed that periphyton acts as biogeochemical regulators, closing the critical N gap in paddy N budgets. This mechanism directly supports the transition toward circular low-loss systems and offers a powerful tool for improving the sustainability of rice-production systems [42,43].

**Figure 5.**
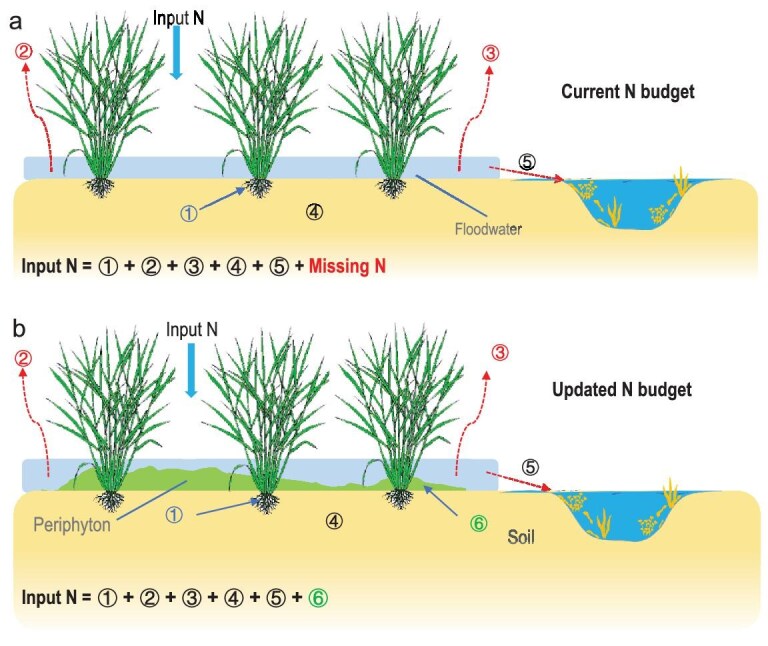
Schematic diagram of N budgets in paddy fields (a) without and (b) with consideration of periphyton. (a) Conventional N-budget model showing a persistent ‘missing N’ gap. Approximately 4%–22% of applied N remains unaccounted for, representing the difference between total inputs and measured outputs. (b) Revised N budget incorporating periphyton-mediated N sequestration, which closes the mass balance gap and results in a complete agroecosystem N budget. Inputs include fertilizer and nonfertilizer N sources (totaling 115%–152%). The N outputs are now fully accounted for across six pathways: i. rice uptake (15%–48%); ii. nitrification/denitrification (14%–45%); iii. ammonia volatilization (8%–32%); iv. soil residual N (12%–38%); v. runoff and leaching (1%–13%); vi. periphyton-regulated N sequestration (6%–24%, this study); missing N: 4%–22% [17–31].

Our comprehensive assessment showed that periphyton sequestered 0.8 Tg N yr⁻^1^ (Equation S6), accounting for 12% of China’s rice N inputs (Fig. 1c and Equation S7). To assess the global significance of periphyton-mediated N sequestration, we compared our findings with detailed N budgets constructed from a comprehensive global inventory of paddy systems [17–31] (Table 1). These budgets consistently report an unexplained N deficit of 4%–22% (mean 13%) of applied N fertilizer (Table 1 and Fig. 5a), which closely matches the missing fraction in our national survey. This correspondence strongly implicates periphyton as the primary sink for unaccounted N and identifies it as a previously overlooked biogeochemical keystone in paddy N cycling. By integrating periphyton-mediated N fluxes into agroecosystem accounting (Fig. 5b), we presented the first complete N balance for rice paddies, establishing periphyton as a natural buffer in the N cycle.

**Table 1.** Historical N balance in paddy rice systems (1980–2023).

	Pathways	Range (%)	Mean (%)	References
Input	N fertilizer	100	100	Baseline
	Nonfertilizer sources	15–52	36	[17–20]
Output	Rice uptake	15–48	34	[19–22]
	Nitrification/denitrification	14–45	33	[20,23–26]
	NH_3_ volatilization	8–32	21	[27–30]
	Residual in soil	12–38	26	[19,24,31]
	Runoff and leaching	1–13	7	[21,22,24,25]
	Unaccounted for	4–22	13	[19,20,22–24,26,29,30]
	N in periphyton	6–24	12	This study

Summary of major N-input and N-output pathways, including ranges, mean contributions and corresponding references. Nonfertilizer inputs include wet and dry deposition, irrigation and biological N fixation. Input and output data were compiled from [14–21,35–38,40,41].

Our findings revealed distinct biogeographical patterns in N sequestration: provincial periphyton N contents ranged from 6.9 ± 0.9 to 15.1 ± 1.6 g kg^−1^, while total N loads increased from (1.7 ± 0.8) × 10^7^ kg in temperate zones to (17.0 ± 6.6) × 10^7^ kg in subtropical regions (Fig. 1b), highlighting the regulatory influence of climate. This pattern likely reflects stronger environmental constraints on N accumulation in cooler regions with pronounced diel temperature fluctuations than in warmer areas, in which faster periphyton biomass turnover may enhance N-sequestration efficiency [44–47].

Nearly 70% of the periphyton-sequestered N replenished the residual soil N pools (Fig. 4), establishing a circular N economy in rice farming systems. We estimate that harnessing periphyton-sequestered N could potentially reduce fertilizer N inputs by 0.2 Tg N yr⁻^1^ (derived from 12% mean sequestration × China’s annual rice N use). The NH_4_⁺-centric storage (Fig. 3) further positioned periphyton as a natural infrastructure for sustainable intensification [41].

Despite the recognized role of periphyton in paddy N cycling, two critical knowledge gaps must be addressed to harness their full potential for improving N-use efficiency. First, the molecular mechanisms governing ammonium NH_4_⁺ release under thermal stress remain poorly understood, necessitating high-resolution metagenomic and transcriptomic analyses to identify the key microbial taxa and functional genes driving temperature-sensitive N turnover [48,49]. Second, the agronomic strategies for synchronizing periphyton-mediated N release with rice N demand remain unknown. Because periphyton acts as a transient N reservoir, storing applied fertilizer N and releasing it gradually through biomass decay, failure to align this release window with critical crop-growth stages may lead to volatilization or leaching losses [41]. Developing management approaches, such as manipulating the water regime, shading or biofilm composition to extend the retention time or delay mineralization, could substantially enhance fertilizer recovery rates in rice systems. Thermal-response algorithms, empirically validated by using our cross-climate ^15^N-tracing approach, could further help enable the precise synchronization of fertilizer applications.

In summary, our work identified periphyton as a major, previously unaccounted-for sink for N in rice paddies, capable of closing the long-standing missing N gap in paddy N budgets. By integrating periphyton-mediated fluxes, we provided the first complete national-scale N balance for rice production and highlighted its potential to reduce fertilizer requirements. Realizing this potential will require resolving key mechanistic uncertainties and developing management practices that align N release with crop demand, positioning periphyton as both a natural buffer and a practical lever for sustainable intensification in flooded agroecosystems.

## CONCLUSION

We identified periphyton as a previously unrecognized yet critical component of the N cycle in rice paddies. Through nationwide field surveys and ^15^N-isotope-tracing experiments, we demonstrated that periphyton immobilizes a substantial fraction of applied N fertilizer and subsequently redistributes it among the soil, gaseous and residual pools. This discovery resolved long-standing discrepancies in paddy N budgets by identifying periphyton as a transient but pivotal N reservoir. These findings close the missing N gap in agroecosystem mass balances and highlight periphyton-mediated buffering as a promising nature-based strategy for improving N-use efficiency and sustainability in global rice-production systems.

## METHODS

Detailed descriptions of all methods and materials are presented in the Supplementary material.

## Supplementary Material

nwag016_Supplemental_File

## Data Availability

Supplementary information is available in the online version of the manuscript. Correspondence and requests for materials should be addressed to Yonghong Wu (yhwu@issas.ac.cn).
