# Supplementary material for: Periphyton closes the nitrogen budget gap in rice paddies
Source: Natl Sci Rev. 2026 Jan 13;13(3):nwag016. doi: 10.1093/nsr/nwag016 (PMC12887296; doi:10.1093/nsr/nwag016)
Supplement: nwag016_Supplemental_File [file nwag016_supplemental_file.docx]

Supplementary Materials for

**Periphyton closes the nitrogen budget gap in rice paddies**

Pengfei Sun^a,b,c^, Yonghong Wu^a,b*^, Yin Chen^c,d^, Jan Dolfing^e^, Bruce E. Rittmann^f^ & Kees Jan van Groenigen^g^

*^a^ State Key Laboratory of Soil and Sustainable Agriculture, Institute of Soil Science, Chinese Academy of Sciences, Nanjing 211135, China*

*^b^ University of Chinese Academy of Sciences,* *Nanjing 211135, China*

*^c^ School of Life Sciences, University of Warwick, Coventry CV4 7AL, UK*

*^d^ School of Biosciences, The University of Birmingham, Birmingham B15 2TT, UK*

*^e^ Faculty of Energy and Environment, Northumbria University, Newcastle upon Tyne NE1 8QH, UK*

*^f^ Biodesign Swette Center for Environmental Biotechnology, Arizona State University, Tempe AZ 85287-5701, USA*

*^g^ Department of Geography, Faculty of Environment, Science and Economy, University of Exeter, Exeter EX4 4QJ, UK*

***Corresponding author**:

Dr. Yonghong Wu

Tel: +86 2586881330; E-mail: [yhwu@issas.ac.cn](mailto:yhwu@issas.ac.cn)

**SUPPLEMENTARY CALCULATIONS**

**Estimating periphyton N stocks in paddy fields at large scales**

The total N stock loadings by periphyton at provincial and national scales (W_TN_) were calculated as follows:

W_TN_ = *B_P_* × *C_N_* × *A*  Eq. S1

where *B_P_* is the periphyton biomass in the paddy fields (kg DW/ha), *N_P_* is the N content in the periphyton (g N/kg DW), and *A* is the area of the paddy fields (ha).

We measured periphyton biomass throughout the rice growth period during on-farm ^15^N isotope tracing experiments and used these data to establish a periphyton growth curve in Chinese paddy fields. Based on this curve, the temporal dynamics of the periphytic biofilm biomass were fitted with three growth equations, each corresponding to a major fertilization stage in rice production (basal, tillering, and panicle fertilization). Field observations across provinces consistently revealed distinct biomass pulses following each fertilizer application, thereby justifying the use of a segmented fitting approach.

The adequacy of each fitted equation was evaluated using the coefficient of determination (R^2^), which ranged from 0.71 to 0.99 across the three phases, indicating strong agreement between the fitted curves and the observed data. Using these equations, we calculated daily periphyton biomass production for each growth stage. These curves reproduced the observed biomass dynamics, confirming their suitability for estimating integrated biomass over the entire rice growth cycle.

$B_{P}=\left\{ \begin{aligned} 86.23e^{0.13t}-86.23, 0\leq x\leq10, R^{2}=0.99 \\ 56.92+0.65t-0.03t^{2}, 10<x<45, R^{2}=0.71 \\ 31.62-0.93t+0.0105t^{2}-3.79\times{10}^{-5}t^{3}, 45\leq x\leq120, R^{2}=0.76 \end{aligned} \right.$ Eq. S2

where *x* is the time of rice growth (d), and *B_P_* is the net periphyton biomass production (kg/ha/day).

The average biomass of periphyton in a hectare paddy field across the 120-d rice growth period (∑B*_P,CHN_*) was quantified by integration:

$${\sum B}_{P,CHN}=\int_{0}^{120} B_{P}\mathrm{dt}=\int_{0}^{10} \left( 86.23e^{0.13t}-86.23 \right)\mathrm{dt}+\int_{10}^{45} \left( 56.92+0.65t-0.03t^{2} \right)dt +\int_{45}^{120} (31.62-0.93t+0.0105t^{2}-3.79\times{10}^{-5}t^{3})dt$$

$=3045 g/kg=3.0 t/ha$ Eq. S3

The weighted-average N content in periphyton at the national scale (*N_P’_* ) was calculated from:

*N_P’ =_*$\frac{\sum C_{\check{N}}\times Ai}{\sum Ai} =8.5 g/kg$ Eq. S4

where $N_{\check{P}}$ is the average N content in periphyton in each province (g/kg) and *A_i_* is the rice planting area in each province (ha).

The average N stock in periphyton per hectare of paddy field in China (*N_CHN_*) was calculated as follows:

*N_CHN_* = *B_P,CHN_* × *C_N’_* =$3045.4$× 8.5×10^-3^ = 25.9 kg/ha Eq. S5

The total rice planting area in China for the past three years has remained stable at ~3.0×10^7^ ha; thus, the total N stock loading by biofilms in all the Chinese paddy fields was calculated as follows:

W_TN_ = *N_CHN_*×*A* = 25.9 × 3.0×10^7^ = 0.8 × 10^9^ kg/year = 0.8 Tg/year Eq. S6

Total N fertilizer consumption in China per rice-growing season in 2014 and 2015 was ~6.6 × 10^9^ kg [[1](#_ENREF_1)]. Based on a nationwide survey of 840 paddy fields, we calculated the ratio of N accumulation in periphyton to the total N fertilizer input (*R_TN_*) as follows:

*R_TN_* = (0.8 × 10^9^) ÷ (6.6 × 10^9^) × 100%=12% Eq. S7

The calculated ratio (*R’_TN_*) of N accumulation in periphyton to total N fertilizer consumption for rice in China, based on the results of ^15^N tracing experiments (*Xi*), was calculated as:

*R’_TN_* = $\frac{\sum Xi}{n}$​=14% Eq. S8

**MATERIALS AND METHODS**

**Description of the study areas**. To quantify total N loadings in periphyton at both provincial (tens to hundreds of kilometers) and national (up to 3,600 km) scales, we sampled 840 sites across 21 regions in 10 provinces of China from 2016 to 2019 (Supplementary Fig. S1A). Sampling was conducted during the early rice-growing season, specifically within 7–15 days after transplanting. China’s paddy fields are broadly distributed across six major geographical regions [[2](#_ENREF_2)], and the 21 selected rice-planting regions collectively represent more than 93% of the country's total rice cultivation area. Detailed information on each sampling region, including sampling dates, number of sampling sites, and corresponding years, is provided in Supplementary Table 2.

**Sample collection.** At each sampling site, one sample each of periphyton, floodwater, and soil was collected. Briefly, approximately 50 g (wet weight) of periphyton was gently scraped from the soil surface using a sterilized stainless-steel knife (Supplementary Fig. S1B) at a 10°–20° angle, and all visible soil clods were removed. Periphytons form a cohesive gelatinous layer (typically 1–3 mm thick) that is distinct from the underlying soil. After periphyton removal, underlying soil samples (0–20 cm) were collected separately with an auger to avoid cross-contamination. Floodwater samples were collected in sterile glass bottles. Soil samples (100 g), excluding surface periphyton, were also taken from 0–20 cm depth with a soil auger and sealed in plastic sampling bags. All samples were transported to the laboratory on ice.

**Periphyton biomass determination.** To quantify the periphyton biomass at each site, three replicate 100 cm^2^ (10 × 10 cm) plots were marked within each paddy field. Periphytons from each plot were collected as described above and transported to the laboratory on ice. In the laboratory, each sample was transferred to an aluminum weighing dish and dried at 60 °C to a constant weight. Biomass values (g per 100 cm^2^) were then converted to per-hectare estimates (kg per ha) to standardize comparisons across sampling locations.

**On-farm** **^15^N isotope tracing experiments.** To quantify the amount of nitrogen fertilizer regulated by periphyton, three on-farm ^15^N isotope tracing experiments were conducted in 2019 across distinct climatic zones: temperate, subtropical, and tropical (Fig. S2). One experiment was established in each climate zone, and within each farm, three replicate plots were set up to capture within-site variability. Accordingly, results for each zone represent the mean of three replicate plots.

All experiments followed local fertilization practices to ensure normal rice growth and yield. Potassium (K) fertilizer was not required in Ledong (tropical zone), but was applied in Jurong (subtropical zone) and Shenyang (temperate zone). Nitrogen (N) and phosphorus (P) fertilizers were required in all sites, though application rates varied by region. Site-specific quantities of ^15^N-labeled urea (10.11 atom% ^15^N) and P_2_O_5_ were determined based on local agronomic recommendations.

Owing to climatic differences, rice growth duration varied across sites. Periphyton samples were collected at fixed intervals, resulting in more sampling events in Shenyang, which had the longest growing season. Further details on the three field experiments are provided below.

*Tropical field experiment.* The tropical field experiment was conducted in Ledong, Hainan Province, China (18°45′N, 109°10′E) during the 2019 rice-growing season. The region has a tropical monsoon climate, with an average annual temperature of 24 °C and rainfall of approximately 1600 mm. The test field contained sandy soil.

Both experimental and control plots (1 × 1 m) were set up in triplicate. Each plot received 37.5 g of labeled urea and 37.5 g of P_2_O_5_ as basal fertilizer on day 0 (7 March). Then, 18.75 g labeled urea was applied 10 d after transplanting (tillering fertilizer, 17 March). Finally, 18.75 g of labeled urea was applied 30 d after rice transplantation (panicle fertilizer, 17 April). Transplanting was performed on 17 March.

Periphyton samples were collected on 28 March, 11 April, 21 April, 1 May, 11 May, and 21 May during the rice growth period. ^15^N content in the sampled periphyton was measured to calculate the proportion of input fertilizer-N sequestrated by periphyton. In addition, the periphyton biomass was also determined at each sampling event using the method described above to construct the periphyton growth curves.

To quantify the partitioning of periphyton-sequestered ^15^N (soil return, NH_3_ volatilization, denitrification, and periphyton residuals), a paired experimental design was used: terbutryne-treated plots (1 × 1 m, periphyton removal) versus untreated controls (natural biofilm growth). Differences in isotopic enrichment between treatments allowed compartment-specific attribution of ^15^N fluxes.

Soil-returned N was calculated as δ^15^N_soil,t_ – δ^15^N_soil,t₀_ (t = sampling date; t_0_ = day 1 post-transplant). Following the approach of our previous work [[3-5](#_ENREF_3)], periphyton‐associated residual N was quantified from the harvested biomass at each sampling point. NH_3_ volatilization was measured continuously for 10 d following fertilizer application using an NH_3_ volatilization field sampler [[5](#_ENREF_5), [6](#_ENREF_6)]. Seasonal NH_3_ fluxes were extrapolated by integrating these daily fluxes during the post-fertilization period. Denitrification was estimated from N_2_O emissions measured at 5-day intervals using the static chamber technique [[7](#_ENREF_7)]. Here, “denitrification” is used operationally to denote gaseous N losses measured as N_2_O. This is based on our previous experiments, which showed that when ^15^N_2_O, ^15^NH_4_⁺-N, or ^15^NO_3_⁻-N were provided as sole N sources, no N_2_ was produced. Residual periphyton N at harvest was calculated by multiplying the biomass (refer to *Periphyton biomass determination*) of periphyton by its N concentration.

To quantify how periphyton influences N redistribution pathways, we employed differential ^15^N tracing based on the following paired experimental plots: 1) ^15^N_control_: isotopic measurements from plots with naturally growing periphyton*.* 2*)* ^15^N_treatment_: Isotopic measurements from plots where periphyton was removed by terbutryn. The periphyton-mediated contribution to each pathway was calculated as: Δ^15^N_pathway_= ^15^N_control_ - ^15^N_treatment_ for each compartment (soil, NH_3_, or N_2_O). This differential approach isolates N fluxes that are specifically attributable to periphyton activity by removing background effects.

*Temperate field experiment*. This field experiment was conducted during the 2019 rice-growing season at the National Field Observation and Research Station of Agro-ecosystems in Shenyang, Liaoning Province, China (41°31′N, 123°24′E). The site has a temperate semi-humid continental monsoon climate with an average annual temperature of 7.5 °C and precipitation of 650 mm. The soil type was brown aquic.

The experimental design mirrored that of the tropical site in Ledong, with experimental and control plots (1 × 1 m) used to track the fate of N in the periphyton. All fertilizers followed local agronomic practices. The rice was transplanted on 4 June. Basal fertilization, applied on 3 June, included 14.4 g of ^15^N-labeled urea, 6.6 g of P_2_O_5_, and 9.4 g of K_2_O. Subsequently, 7.2 g of ^15^N-labeled urea was applied as a tillering fertilizer on 13 June, and 2.4 g as a panicle fertilizer on 3 July. Periphyton samples were collected throughout the rice growth period on the following dates: 6, 7, 9, 10, and 20 June; 11, 13, and 17 July; 25 July; 27 and 29 August; and 5 and 20 September. For each sampling event, ^15^N content in both the periphyton and paddy soils was measured. In addition, periphyton biomass was determined at each sampling event using the method described above to establish the periphyton growth curve.

*Subtropical field experiment.* The field experiment was conducted in Jurong, Jiangsu Province, China (31°58′12″N, 119°21′E) during the 2019 rice-growing season. The site has a subtropical monsoon climate and follows a rice–wheat rotation. The average annual temperature and precipitation were 15.2 °C and 1050 mm, respectively. The soil at our site is classified as yellow-brown. Experimental and control plots (1 × 1 m) were established following the design used by Ledong and Shenyang to measure the fates of N regulated by periphyton: return to soil and loss via ammonia volatilization. Fertilizer applications were as follows: 37.5 g ^15^N-labeled urea, 37.5 g P_2_O_5_, and 37.5 g K_2_O on 1 July; 22.5 g ^15^N-labeled urea as tillering fertilizer on 12 July; and 22.5 g ^15^N-labeled urea as panicle fertilizer on 2 August. Periphyton samples were collected on 2, 4, 6, 8, 24, 27, and 30 July and 28 August, and their ^15^N contents were analyzed. In addition, the periphyton biomass was determined at each sampling event using the method described above to establish the periphyton growth curve.

The proportion of urea-^15^N regulated by the periphyton (N_P_/N_F_(%)) at all three sites was calculated as follows:

$N_{P}/N_{F}(\%)=N_{S}/N_{U}\times100\%$ Eq. S9

where N_P_/N_F_(%) is the proportion of urea-^15^N regulated by the periphyton (%), N_S_ is the amount of urea-^15^N in each periphyton sample (kg), and N_U_ is the quantity of urea-^15^N used for each farm experiment (kg). Finally, using the three newton fertilization periods (basal, tillering, and panicle) as time nodes, we determined soil ^15^N content, NH_3_ volatilization flux, and denitrification potential for each field experiment. Based on these measurements, we quantified the dynamic fate of regulated N in the periphyton throughout the rice growth period.

**Sample pretreatment and analysis.** Total nitrogen (TN) concentrations in the periphyton and soil were determined following Bao [[8](#_ENREF_8)]. Periphyton samples were placed in aluminum trays, oven-dried at 60 °C to constant weight, and ground. Subsamples (0.5 g dry weight) were digested in 10 mL nitric acid at 90 °C for 1 h. After cooling, 3 mL hydrogen peroxide was added, and the mixture was maintained at 90 °C for 1 h. The temperature was then increased to 95 °C, and the solution was reduced to ~2 mL by evaporation. After cooling, the samples were filtered through a medium-speed filter paper (FFT08, Beyotime, China), diluted with ultrapure water to 25 mL, and left to stand for 24 h. N concentrations were measured using a flame photometer (Sherwood M410, UK). Soil samples (0.5 g dry weight) were processed identically prior to TN determination.

The TN, NH_4_^+^-N, and NO_3_^-^-N concentrations in the floodwater and water samples collected in the laboratory experiment were measured using a flow analyzer (San++System, SAKLAR, Netherlands). ^15^N content in the periphyton and paddy soils was detected using an isotope ratio mass spectrometer 253 plus (MAT 253 plus, Thermo Fisher Scientific, Germany). The N_2_O concentrations collected via static chambers were determined by GC–µECD (GC-MS; Agilent Technologies) calibrated with certified NOAA/BOC standards, and ^15^N_2_O enrichment was quantified using a continuous-flow isotope ratio mass spectrometer (CF-IRMS; Delta V Plus, Thermo Fisher Scientific) coupled to a PreCon system. ^15^N_2_ production was determined with the N₂:Ar ratio method using elemental analysis–CF-IRMS (Flash EA 1112 coupled to Delta V Plus), where O_2_ was removed by hot-copper reduction and N_2_ isotopologues (m/z 28, 29, 30) were measured relative to air standards to calculate atom% ^15^N_2_ excess [[9](#_ENREF_9)]. ^15^NH_3_ volatilization was quantified by isotopic analysis using a continuous-flow isotope ratio mass spectrometer (Delta V Advantage, Thermo Scientific).

**Systematic literature review for N fates in paddy fields.** Comprehensive systematic searches were conducted across Web of Science Core Collection, Google Scholar, and PubMed (1980–2023) using the Boolean query: (("N fate" OR "N budget" OR "nitrogen balance" OR "N pathway") AND ("rice paddy" OR "paddy field" OR "flooded rice") AND ("unaccounted N" OR "missing N" OR "N deficit")). Supplementary keywords included N cycling efficiency, ^15^N tracing, biogeochemical flux, fertilizer residual, and N recovery*.* Both input and output pathway data and unaccounted N in global rice systems were synthesized from studies spanning nearly 40 years.

**Statistical analyses.** All statistical procedures were conducted using the SPSS software (version 16.0; SPSS Inc., Chicago, IL, USA). Figures were plotted using the Origin 8.5 software (OriginLab Inc., Massachusetts, USA). All calculations are described in the Supplementary Information.

**SUPPLEMENTARY FIGURES**


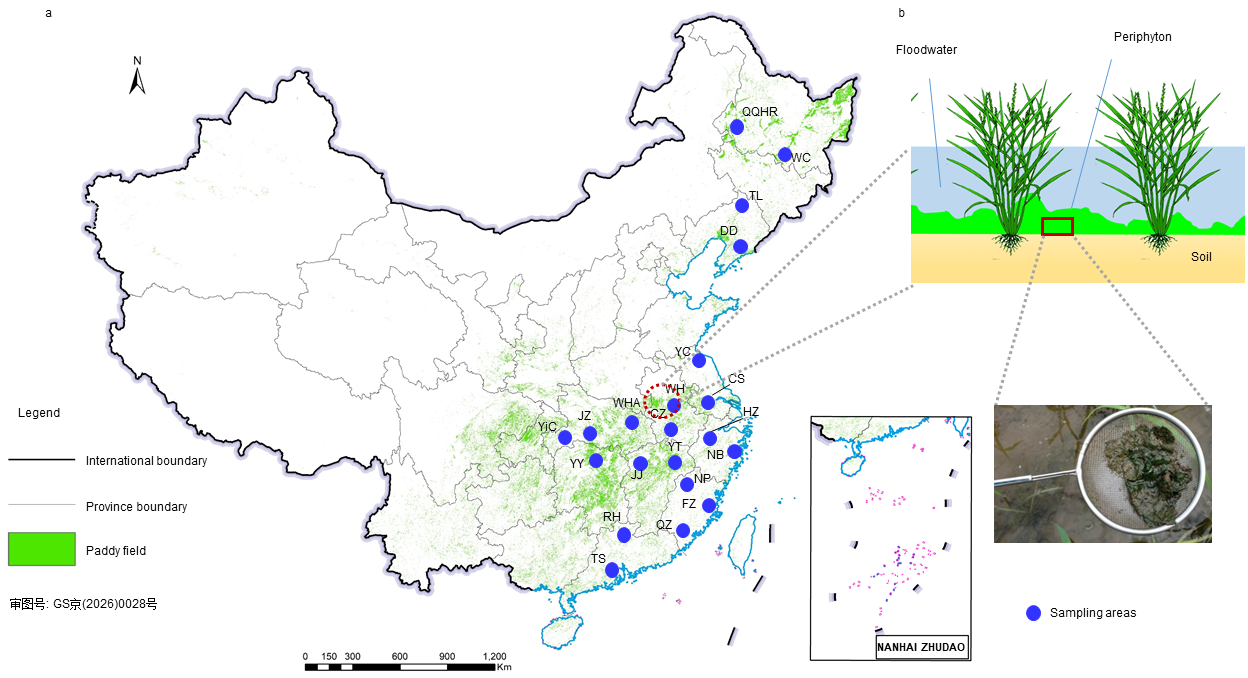


**Fig. S1 Map of the 21 sampling areas located in different rice growing areas across China (a) and the diagram of collecting periphytons (b).** Sampling areas along with the hydraulic gradient (from western to eastern China) are: YiC: Yichang, YY: Yueyang, JZ: Jingzhou, JJ: Jiujiang, WHA: Wuhan, CZ: Chizhou, WH: Wuhu, YC: Yancheng, and CS: Changshu; Sampling areas along with the thermal gradient (from southern to northern China) are: TS: Taishan, RH: Renhua, QZ: Quanzhou, FZ: Fuzhou, NP: Nanping, YT: Yingtan, NB: Ningbo, HZ: Hangzhou, DD: Dandong, TL: Tieling, WC: Wuchang, QQHR: Qiqihar.

**
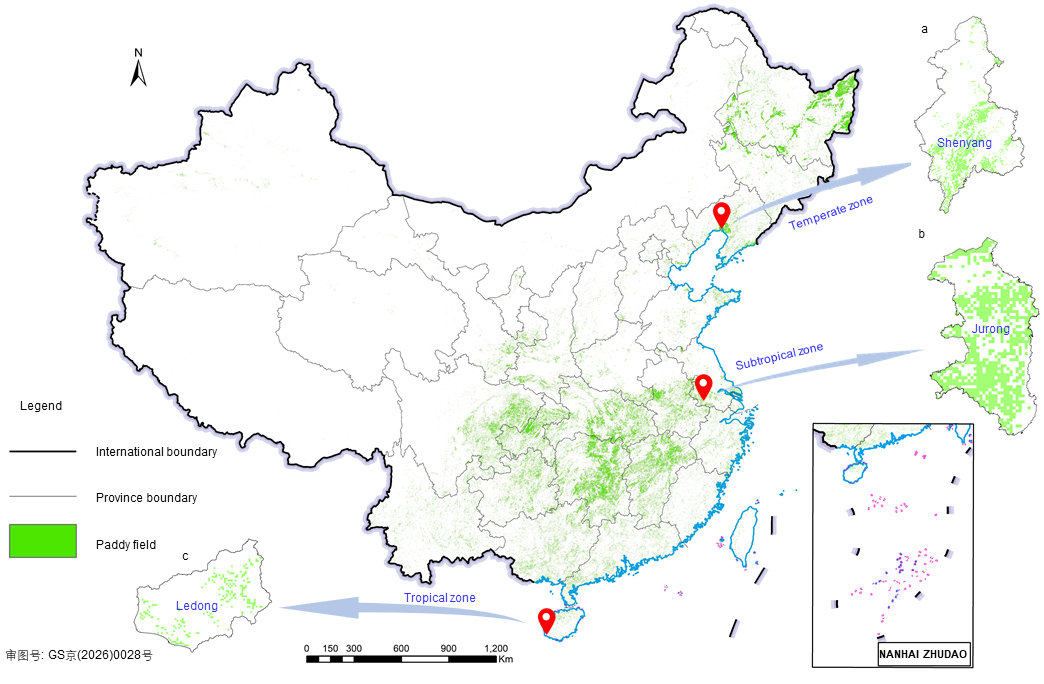
**

**Fig. S2 Map of the three on-farm experiments located in the three temperature zones.** a: temperate zone (Shenyang, Liaoning province); b: subtropical zone (Jurong, Jiangsu province); and c: tropical zone (Ledong, Hainan province).


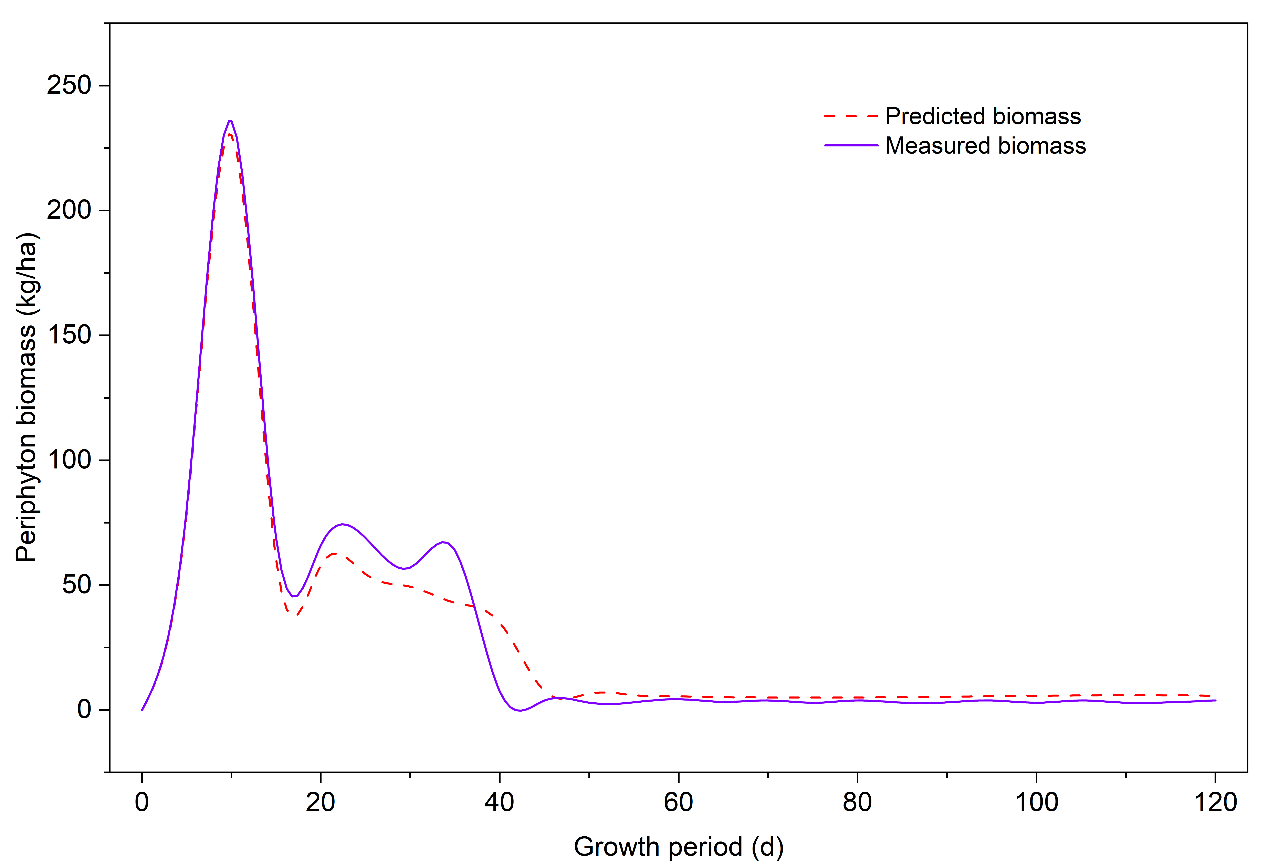


**Figure S3.** Comparison of the measured growth curve with the predicted curve based on the derived equations. The close agreement between the two curves demonstrates the accuracy of the equations we developed.

**SUPPLEMENTARY TABLES**

**Table S1.** Rice planting areas and average fertilizer-N application rates for rice production across Chinese provinces, as compiled from the National Bureau of Statistics of China [[1](#_ENREF_1)] .

| Province | Average N fertilizer application (kg ha^-1^) | Rice planting area (kha, 2016-2019) |
| --- | --- | --- |
| Zhejiang | 270 | 628±16 |
| Anhui | 225 | 2549±40 |
| Liaoning | 195 | 491±12 |
| Hunan | 180 | 4095±199 |
| Jiangsu | 300 | 2223±31 |
| Fujian | 166 | 620±14 |
| Heilongjiang | 250 | 3867±82 |
| Guangdong | 150 | 1798±9 |
| Hubei | 210 | 2351±45 |
| Jiangxi | 225 | 3453±81 |

**Table S2.** Summary of periphyton sample collection across 21 sampling areas in 10 provinces, including coordinates, number of sites sampled from 2016–2019, and sampling dates.

| Provinces | Sampling areas | Longitude | Latitude | Number of sites sampled | | | | Sampling time*  (± 2 d) |
| --- | --- | --- | --- | --- | --- | --- | --- | --- |
|  |  |  |  | 2016 | 2017 | 2018 | 2019 |  |
| Guangdong | Taishan | 22.25 | 112.79 | 10 | 10 | 10 | 10 | 19 April  17 April |
|  | Renhua | 25.09 | 113.75 | 10 | 10 | 10 | 10 |  |
| Fujian | Quanzhou | 24.89 | 118.61 | 10 | 10 | 10 | 10 | 13 May  12 May  14 May |
|  | Fuzhou | 26.05 | 119.27 | 10 | 10 | 10 | 10 |  |
|  | Nanping | 27.33 | 118.12 | 10 | 10 | 10 | 10 |  |
| Jiangxi | Yingtan | 28.24 | 117.04 | 10 | 10 | 10 | 10 | 9 July  10 July |
|  | Jiujiang | 29.73 | 115.99 | 10 | 10 | 10 | 10 |  |
| Hubei | Yichang | 30.53 | 111.43 | 10 | 10 | 10 | 10 | 3 June  4 June  2 June |
|  | Jingzhou | 30.35 | 112.19 | 10 | 10 | 10 | 10 |  |
|  | Wuhan | 30.48 | 114.32 | 10 | 10 | 10 | 10 |  |
| Hunan | Yueyang | 29.46 | 113.01 | 10 | 10 | 10 | 10 | 25 May |
| Anhui | Wuhu | 31.34 | 118.39 | 10 | 10 | 10 | 10 | 20 June  19 June |
|  | Chizhou | 30.69 | 117.57 | 10 | 10 | 10 | 10 |  |
| Zhejiang | Hangzhou | 30.21 | 120.21 | 10 | 10 | 10 | 10 | 9 July  10 July |
|  | Ningbo | 29.90 | 121.84 | 10 | 10 | 10 | 10 |  |
| Jiangsu | Changshu | 31.65 | 120.75 | 10 | 10 | 10 | 10 | 3 July  2 July |
|  | Yancheng | 33.20 | 120.50 | 10 | 10 | 10 | 10 |  |
| Liaoning | Dandong | 40.14 | 124.40 | 10 | 10 | 10 | 10 | 15 July  16 July |
|  | Tieling | 42.55 | 124.16 | 10 | 10 | 10 | 10 |  |
| Heilongjiang | Wuchang | 44.93 | 127.17 | 10 | 10 | 10 | 10 | 12 July  13 July |
|  | Qiqihar | 47.16 | 123.82 | 10 | 10 | 10 | 10 |  |

REFERENCES

1. China NBoSo. <http://data.stats.gov.cn/>.

2. Mu T, Wu T, Zhou T *et al.* Geographical variation in arsenic, cadmium, and lead of soils and rice in the major rice producing regions of China. *Sci Total Environ* 2019; **677**: 373-381.

3. Xia Y, She D, Zhang W *et al.* Improving denitrification models by including bacterial and periphytic biofilm in a shallow water-sediment system. *Water Res Res* 2018; **54**: 8146-8159.

4. Sun P, Chen Y, Liu J *et al.* Periphytic biofilms function as a double-edged sword influencing nitrogen cycling in paddy fields. *Environmental Microbiology*. 2022; **24**(12): 6279-6289.

5. Zhao J, Han J, Sun P, Wu Y. Effect and mechanism of periphyton affecting ammonia volatilization in paddy field. *Acta Pedol Sin* 2021; **58**: 1267-1277.

6. Kissel DE, Brewer HL, Arkin GF. Design and test of a field sampler for ammonia volatilization. *Soil Sci Soc Am J* 1977; **41**: 1133-1138.

7. Shan J, Sanford RA, Chee‐Sanford J *et al.* Beyond denitrification: the role of microbial diversity in controlling nitrous oxide reduction and soil nitrous oxide emissions. *Global Change Biol* 2021; **27**: 2669-2683.

8. Bao SD. Soil and agricultural chemistry analysis. *Agriculture Publication*. 2000: 355-356.

9. Si Y, Zhu Y, Sanders I *et al.* Direct biological fixation provides a freshwater sink for N_2_O. *Nat Commun* 2023; **14**: 6775.
